# Comparative Evaluation of Temperature, Bottle Type, and Amphotericin B on Fungal Detection in Corneal Preservation Media Using the BACT/ALERT System^®^

**DOI:** 10.3390/microorganisms13112562

**Published:** 2025-11-10

**Authors:** Aleix Fernández, Margarita Blanco, Cristina Garcia, Mariama Jallow, Sara Monge, Mar López, Marina Hortolà, Elba Agustí, Anna Vilarrodona, Gloria Soria

**Affiliations:** 1 Microbiology Laboratory, Banc de Sang i Teixits, 08005 Barcelona, Spain; 2 Barcelona Tissue Bank, Banc de Sang i Teixits, 08005 Barcelona, Spain; 3 Donació i Trasplantament d’Òrgans, Teixits i cèl·Lules, Vall d’Hebron Institut de Recerca (VHIR), 08035 Barcelona, Spain; 4 Grup de Medicina Transfusional, Vall d’Hebron Institut de Recerca (VHIR), 08035 Barcelona, Spain

**Keywords:** cornea, transplantation, fungal detection, BACT/ALERT^®^, iFA Plus, iLYM, yeasts, filamentous fungi, 32 °C, 37 °C

## Abstract

Fungal contamination of donor corneas, although rare, constitutes a serious infection risk to recipients. Therefore, microbiological quality control with methods optimized for fungal detection is crucial in eye banks to detect and discard contaminated corneas. This study aimed to compare the performance of different BACT/ALERT^®^ bottles (iFA Plus and iLYM) incubated at different temperatures (32 °C and 37 °C) when corneal preservation media, with or without amphotericin B, were cultured. A total of 6 culture conditions were compared by inoculation of fewer than 100 colony-forming units of 18 fungi (10 yeasts and 8 filamentous fungi). iFA Plus bottles incubated at 32 °C reliably detected 94.4% of fungi, regardless of amphotericin B presence, while iLYM bottles detected 77.8% in the absence of amphotericin B and 72.2% in its presence. At 37 °C, iFA Plus fungal detection decreased to 61.1%. iFA Plus bottles incubated at 32 °C provided the highest detection rates, effectively neutralizing the effect of amphotericin B and enabling recovery of both yeasts and filamentous fungi, except for *Cladosporium*. Our findings support that the optimal strategy for cornea sterility testing is incubating iFA Plus bottles at 32 °C for optimal fungal recovery, while maintaining iFN Plus at 37 °C to allow timely detection of anaerobic bacteria.

## 1. Introduction

Corneal transplantation, or keratoplasty, is a surgical procedure that replaces damaged or non-functional corneal tissue with healthy donor tissue. This treatment is essential for managing various ocular conditions affecting the cornea’s shape, transparency, or structural integrity—such as degenerative diseases, hereditary dystrophies, trauma, or infections—making it one of the most performed transplants worldwide [1]. Different transplantation modalities exist depending on the type and location of corneal damage. The classical technique, penetrating keratoplasty (PK), involves replacing the full thickness of the cornea but presents drawbacks such as structural graft failure and a higher risk of immune rejection [2]. Recently, lamellar keratoplasties—both anterior (Deep anterior lamellar keratoplasty-DALK) and posterior (Descemet’s stripping automated endothelial keratoplasty-DSAEK, Descemet Membrane Endothelial Keratoplasty-DMEK)—have gained prominence by enabling selective replacement of damaged corneal layers while preserving healthy recipient tissue, resulting in faster visual recovery and reduced rejection rates [3,4].

The reported incidence rates of post-keratoplasty infection differ considerably from 0.02 to 13% depending on transplant type and geographic region, with a preponderance of bacteria over fungi and *Staphylococcus* spp. as the leading pathogen [5,6,7,8,9,10]. Fungal infections account for less than 20% of cases and occur more often in warm and humid environments, with *Candida* spp. being the most prevalent pathogen, although *Fusarium* spp. and *Aspergillus* spp. infections have also been reported [5]. Infection can lead to serious complications, including graft rejection and failure. It requires antimicrobial treatment and even surgical interventions in medically refractory cases and may result in reduced visual acuity or even blindness of the patient [5]. Mycoses are among the most difficult corneal transplant infections to treat [10,11].

Microbial contamination of donor tissues stands as an important factor among others contributing to infection, such as host health status, topical corticosteroid use, suture-related problems or previous corneal infection [6]. Although its usefulness is controversial because there is no clear correlation to infection [12], corneoscleral rim cultures performed with surplus tissue after surgery show contamination rates ranging from 0.37% to 14% [7,12,13,14,15,16], with bacteria being more prevalent than fungi, and *Staphylococcus* spp. and *Candida* spp. the main representatives, respectively. Some studies have shown a much higher agreement between rim culture and fungal infections when compared with bacterial infections [16,17] and propose their use to guide prophylactic treatment.

Cornea contamination may occur during procurement, handling or storage [9,18,19] since a variety of microorganisms are present on the eyelids and conjunctival sacs of normal eyes [9]. Different factors such as the donor’s cause of death, antibiotic use, and length of hospital stay prior to death may influence the microbial load of the ocular surface and increase the risk of contamination [20,21]. Additionally, the prevalence and microbial spectrum vary depending on the geographic region and the specific practices of each eye bank [18,19].

Furthermore, microbiological controls are performed at different time points during storage and contaminated corneas are discarded. These controls have shown annual contamination rates of corneal preservation media ranging from 1.1% to 7.2% with bacterial contamination being the most prevalent (78% to 97% of positive cultures). Gram-Positive bacteria, including *Staphylococcus* spp., *Corynebacterium* spp., and *Bacillus* spp., followed by Gram-Negative bacteria such as *Pseudomonas aeruginosa*, *Enterobacter* spp., and *Klebsiella* spp., and fungi such as *Candida* spp. were the most frequent isolates [9,19,20,21,22,23,24,25].

Therefore, the prevention of infection resulting from microbial contamination of donor tissues is a major challenge. Eye banks have instituted several measures, such as aseptic techniques during corneal tissue procurement and disinfection protocols with povidone-iodine, to try to decrease contamination of donor corneal tissue. Storage conditions are also a critical factor. Hypothermia (2–8 °C) is the most commonly applied method of storage, which allows storage for 7–14 days. Organ culture (28–37 °C), which extends storage time to 4 weeks, is used widely in European eye banks [26]. Warmer temperatures are more favorable to microbial growth, but extended storage time increases the probability to detect contamination when cultures are routinely performed [9], which is one of the most crucial steps for tissue stored by organ culture. This practice may contribute to the significantly reduced rates of positive postoperative rim cultures with organ culture storage [9].

Storage solutions typically contain antibacterial agents (gentamicin and streptomycin) [27,28]. However, nutrient-rich conditions can also support the growth of yeasts and filamentous fungi [29]. At this point, amphotericin B has emerged as a valuable additive to preservation media [30,31], as it binds to ergosterol in the fungal cell membrane via its hydrophobic domains, forming multimeric pores in the lipid bilayer that destabilize the membrane, increase ionic permeability, disrupt cellular homeostasis, and ultimately cause fungal cell death [31,32,33,34]. However, while amphotericin B is highly effective against some yeast species like *Candida albicans* and *Candida tropicalis*, its activity is more limited against certain filamentous fungi, such as *Fusarium* spp. and *Scedosporium* spp., as well as some resistant non-*albicans Candida* strains [35], and it is dependent on the concentration included in the media.

In summary, fungal infection after corneal transplantation is a rare, yet potentially devastating, postoperative complication and its incidence has been growing in recent years with the rise of lamellar keratoplasty techniques such as DSAEK and DMEK, which require tissue processing. Eye banks have to be prepared to effectively detect fungal contamination.

At the Blood and Tissue Bank in Barcelona, cornea processing follows the guidelines established by the Guide to the quality and safety of tissues and cells for human application [36] regarding donor selection, procurement, storage, quality control by evaluation of endothelial cell viability via specular microscopy and trypan blue staining, and microbiological testing. Donor corneas undergo a standardized microbiological testing protocol designed to enable early microbial detection and to ensure the safety and quality of corneal grafts. Three different media are used sequentially for their conservation: an extraction medium, employed from tissue retrieval until evaluation; a preservation medium, used to maintain the tissue for up to four weeks; and a transport medium, applied to deswell the tissue prior to transplantation. The extraction medium is incubated at 32 °C for up to 14 days and visually inspected daily for indicators of microbiological contamination, such as turbidity or color change due to medium acidification. Moreover, samples from the preservation and transport media are inoculated into one iFA Plus and one iFN Plus BACT/ALERT^®^ culture bottles (bioMérieux, Marcy-l’Étoile, France) and incubated at 37 °C for 14 days to detect microbial contamination using the BACT/ALERT^®^ 3D system (bioMérieux, Marcy-l’Étoile, France). Endorsed by the European Pharmacopoeia (Ph. Eur.) for sterility testing, the BACT/ALERT is a growth-based automated system that has demonstrated sensitivity equivalent to traditional pharmacopeia methods while markedly reducing detection times [37,38].

However, incubation at 37 °C may not optimally support the growth and detection of certain environmental yeasts and filamentous fungi, which often proliferate more effectively at lower temperatures, such as 32 °C [29,39]. This limitation was clearly demonstrated in a recent case in which a contaminated cornea was transplanted because the system failed to detect *Aspergillus versicolor* in culture bottles incubated at 37 °C [40]. Additionally, the type of culture bottle used can significantly influence detection sensitivity; for instance, iLYM bottles has been reported to enhance the growth of fungal contaminants [29].

Therefore, the purpose of this study was to compare two incubation temperatures (32 °C and 37 °C) and two types of BACT/ALERT^®^ culture bottles (iFA Plus and iLYM) with two types of corneal preservation media (with or without amphotericin B) using low-level inocula (<100 CFU/mL), in accordance with Ph. Eur. [36], with the aim of determining the optimal conditions to detect fungal contamination in corneal preservation media using an automated system.

## 2. Materials and Methods

### 2.1. Fungal Strains

A total of eighteen fungal isolates were analyzed. Sixteen were obtained from routine microbiological controls of corneal preservation media and represented 85.7% (18 out of 21) of the fungal species identified from January 2024 until April 2025, including nine yeasts (*Candida albicans*, *Candida tropicalis*, *Candida parapsilosis*, *Candida glabrata*, *Clavispora lusitaniae*, *Pichia norvegensis*, *Pichia kudriavzevii*, *Wickerhamomyces anomalus*, *Debaryomyces hansenii*) and seven filamentous fungi (*Aspergillus fumigatus*, *Penicillium chrysogenum*, *Arthrinium arundinis*, *Cladosporium sphaerospermum*, *Stachybotrys chlorohalonata*, *Alternaria alternata*, and *Curvularia* sp). All isolates were identified by Matrix-Assisted Laser Desorption/Ionization Time-of-Flight (MALDI-TOF) using the MaldiBiotyper Sirius (Bruker, Bremen, Germany) and were stored at −80 °C with 20% glycerol until use. All contaminated corneas were discarded. Additionally, two reference fungal strains were included, prepared from commercially standardized inocula (BIOBALL^®^, bioMérieux, Marcy-l’Étoile, France): the yeast *Candida albicans* ATCC 10231 and the filamentous fungi *Aspergillus brasiliensis* ATCC 16404.

### 2.2. BACT/ALERT^®^ System and Culture Bottles

Microbiological monitoring was carried out using the BACT/ALERT^®^ 3D system (bioMérieux, Marcy-l’Étoile, France), an automated platform that continuously monitors culture bottles through a colorimetric sensor detecting carbon dioxide (CO_2_) released during microbial growth [41,42,43].

Two types of culture bottles were used: iFA Plus with a maximum incubation time of 14 days, and iLYM with a maximum incubation time of 7 days. The iFA Plus bottles contain an aerobic complex medium with polymeric beads that neutralize residual antimicrobials, supporting the growth of a broad range of aerobic and facultative microorganisms [44]. The iLYM bottles contain an acidic, carbohydrate-enriched medium designed to promote the growth of yeasts, filamentous fungi, and some facultative anaerobes, but not strict anaerobes [45].

### 2.3. Corneal Preservation Media

The suitability of the BACT/ALERT^®^ system was evaluated for six corneal media: Corneal Chamber^®^ (AL.CHI.MI.A, Ponte San Nicolò, PD, Italy) is an extraction medium containing the Eusol-C solution and gentamicin, for corneal storage up to 14 days at 4 °C; Kerasave^®^ (AL.CHI.MI.A, Ponte San Nicolò, PD, Italy) is an extraction medium, containing the Eusol-C solution, gentamicin, streptomycin and amphotericin B at 2.5 μg/mL, for corneal storage up to 30 days at 4 °C; Tissue-C^®^ (AL.CHI.MI.A, Ponte San Nicolò, PD, Italy) is a preservation medium for corneal storage at 31 °C that contains penicillin G, streptomycin and amphotericin B; Carry-C^®^ (AL.CHI.MI.A, Ponte San Nicolò PD, Italy) is a transport medium for corneal deswelling at room temperature, which also contains penicillin G, streptomycin, amphotericin B and dextran; Cornea Max^®^ (Eurobio, Les Ulis, France) is a preservation medium for corneal storage at 31 °C up to 30 days, which is supplemented with amphotericin B at 0.25 μg/mL; Cornea Jet^®^ (Eurobio, Les Ulis, France) is a transport medium for corneal deswelling at room temperature, which is also supplemented with amphotericin B at 0.25 μg/mL.

### 2.4. Sample and Control Preparation

Frozen aliquots of fungal strains were thawed at room temperature and inoculated onto selective solid media: yeast were streaked onto Tryptone Soy Agar (TSA), while filamentous fungi were cultured on Sabouraud Dextrose agar (SDA). Plates were incubated at 32 °C for yeasts and at 22 °C for filamentous fungi until colonies became macroscopically visible.

Unicellular suspensions were prepared in sterile saline, using protocols adapted to microbial type. Yeast colonies were aseptically collected with a Kolle loop and transferred into tubes containing sterile 0.9% NaCl. Sporulated filamentous fungi were overlaid with sterile saline containing 0.05% Tween 20 and gently scraped with a Digralsky loop to release spores. Suspensions were allowed to settle in tubes for 15 min to remove hyphal fragments, and the spore-enriched supernatant was transferred to new tubes for further use.

Microbial concentrations were determined using a Neubauer counting chamber and adjusted to approximately 10^5^ CFU/mL. Suspensions exceeding the target value were diluted with sterile saline, while those below the target were reprocessed to increase the microbial load, ensuring homogeneous suspensions at the desired concentration.

For each fungal species, a dilution series was prepared, and all subsequent inoculations were performed in triplicate. For each replicate, 0.1 mL of a suspension containing fewer than 100 CFU/mL was inoculated into two iFA Plus bottles and one vented iLYM bottle. Inoculum concentrations were confirmed by plate counting.

To compare two media—Corneal Chamber and Corneal Chamber supplemented with amphotericin B (2.5 μg/mL)—0.9 mL of the respective medium was added per bottle, and the procedures were carried out in parallel. One set of iFA Plus and iLYM bottles was incubated at 32 °C, while the remaining set of iFA Plus bottles was incubated at 37 °C, using the BACT/ALERT^®^ system. Positive cultures were subcultured on TSA or SDA and identified by MALDI-TOF.

Each experiment included positive controls, in which microorganisms were inoculated into bottles without corneal media, and negative controls, using sterile saline or uncontaminated medium to verify the absence of contamination.

### 2.5. Data Analysis

Graphs and statistical analysis were performed using GraphPad Prism software version 5.03 (GraphPad Software, San Diego, CA, USA) and Microsoft Excel. Comparisons between experimental conditions—such as bottle type and incubation temperature—were conducted using the paired Student’s *t*-test. Additionally, Fisher’s exact test was applied to assess differences in the distribution of positive and negative outcomes across these parameters. Statistical significance is denoted in figures as: * (*p* ≤ 0.05), ** (*p* ≤ 0.01), *** (*p* ≤ 0.001). Non-significant differences are shown as ns (*p* > 0.05), while NA denotes cases where statistical analysis was not applicable due to absence of replicates.

## 3. Results

### 3.1. Previous Considerations: BACT/ALERT^®^ Performance

#### 3.1.1. Growth Promotion Properties of BACT/ALERT^®^ Bottles

The growth-promoting properties of each batch of culture bottles used for sterility testing were routinely verified by inoculating fewer than 100 CFU of each test microorganism, in accordance with the Ph. Eur. requirements. Aerobic iFA Plus bottles were inoculated with *Staphylococcus aureus*, *Pseudomonas aeruginosa*, *Bacillus subtilis* subsp. *spizizenii*, *Candida albicans*, and *Aspergillus brasiliensis*. Anaerobic iFN Plus bottles were inoculated with *Clostridium sporogenes* and *Bacteroides fragilis*. All bacterial strains were detected within 3 days and all fungal strains within 5 days, complying thus with Ph. Eur. criteria.

iLYM bottles were also evaluated for growth promotion by inoculating fewer than 100 CFU of each control strain. Under these conditions, only *Pseudomonas aeruginosa* and *Candida albicans* were detected by the BACT/ALERT^®^ system, at 27.8 and 27.5 h, respectively. *Aspergillus brasiliensis* was not detected by the system; however, post-incubation visual inspection revealed fungal growth inside the bottles. After consulting with bioMérieux, it was recommended to vent the bottles prior to inoculation in order to introduce oxygen and thereby enhance fungal growth. Following this adjustment, *Aspergillus brasiliensis* was successfully detected within 36 h. Consequently, vented iLYM bottles were considered suitable for the detection of yeasts and filamentous fungi as required by Ph. Eur. requirements.

#### 3.1.2. Method Suitability

In compliance with the Ph. Eur., the suitability of the BACT/ALERT^®^ method for corneal preservation media was demonstrated by evaluating specificity, sensitivity, reproducibility, and robustness.

##### Specificity

No false-positive results were observed during the experiments. Control bottles inoculated with uncontaminated sterile saline or corneal preservation media consistently remained negative throughout the incubation period.

##### Sensitivity

**Figure 1:**
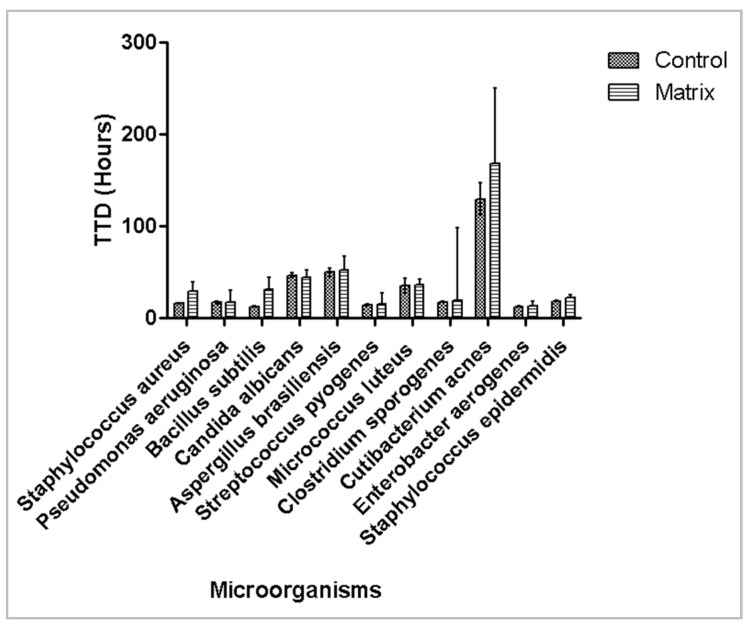
Effect of corneal preservation media on time to detection of microbial growth. Mean time to detection (hours) with 95% confidence interval is plotted.

The 6 corneal preservation media tested were Corneal Chamber, Kerasave, Cornea Max, Tissue-C, Cornea Jet, and Carry-C. As the maximum volume of these media is 100 mL, 1 mL (1%) of each one was inoculated into iFA Plus BACT/ALERT^®^ bottles. Inocula of <100 CFU of each test microorganism were prepared in duplicate, and time to detection (TTD) was recorded. iFA Plus bottles were incubated at 37 °C. All microorganisms were detected within 3 days (minimum TTD: 12.4 ± 0.1 h for *Klebsiella aerogenes*; maximum TTD: 50.3 ± 5.5 h for *Aspergillus brasiliensis*), except for *Cutibacterium acnes*, which required 5.2 days, as shown in Figure 1. Overall, the presence of corneal media did not significantly affect microbial detection compared with controls bottles without media.

##### Effect of Inoculum

**Figure 2:**
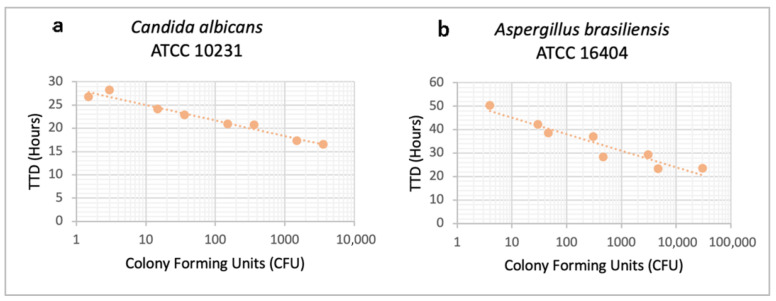
Effect of inoculum on time to detection for (**a**) *Candida albicans* ATCC 10231 and (**b**) *Aspergillus brasiliensis* ATCC 16404. Time to detection (hours) from two independent experiments is plotted for each fungal concentration (Colony Forming Units).

To assess the effect of inoculum size, four serial dilutions of the control strains *Candida albicans* ATCC 10231 and *Aspergillus brasiliensis* ATCC 16404, ranging approximately from 10^4^ to 10^1^ CFU, were inoculated into iFA Plus bottles, and TTD was determined at 37 °C in two independent experiments. As shown in Figure 2, TTD was inversely proportional to inoculum size, and the system was capable of detecting as few as 1.5 CFU of *Candida albicans* ATCC 10231 and 4 CFU of *Aspergillus brasiliensis* ATCC 16404.

##### Reproducibility and Robustness

To evaluate reproducibility and robustness, <100 CFU of the microorganisms specified in the Ph. Eur. were inoculated into iFA Plus bottles and incubated at 37 °C in six independent experiments conducted by different technicians. The mean, standard deviation (SD), and coefficient of variation (CV) of the TTD were calculated. CV values were below 10%, as shown in Table 1, except for *Cutibacterium acnes*. CV ranged from 1.1% for *Enterobacter aerogenes* to 13.1% for *Cutibacterium acnes*.

#### 3.1.3. Effect of Incubation Temperature on Reference Strains

**Figure 3:**
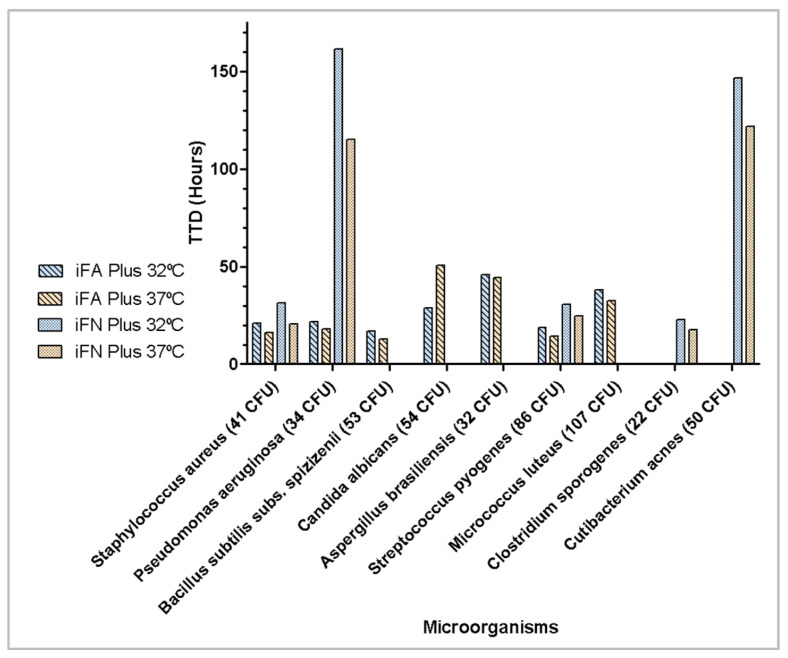
Effect of incubation temperature on time to detection of microbial growth (hours).

To assess the effect of incubation temperature, <100 CFU of the microorganisms recommended by Ph. Eur. were inoculated in iFA Plus bottles (aerobic) or iFN Plus bottles (anaerobic) and incubated at 32 °C and 37 °C. All microorganisms, both bacteria and fungi, were detected within 3 days, except for *Cutibacterium acnes*, which required 5.1 days at 37 °C and 6.1 days at 32 °C, as shown in Figure 3.

### 3.2. Fungal Detection: Comparative Analysis

With the aim of identifying the optimal conditions for fungal detection, two types of culture bottles (iFA Plus and iLYM) and two incubation temperatures (32 °C and 37 °C) were evaluated using eighteen fungal isolates in Corneal Chamber media, with or without amphotericin B supplementation. Detailed graphs for each microorganism are provided in Supplementary Figure S1 and are summarized in Table 2.

**Figure 4:**
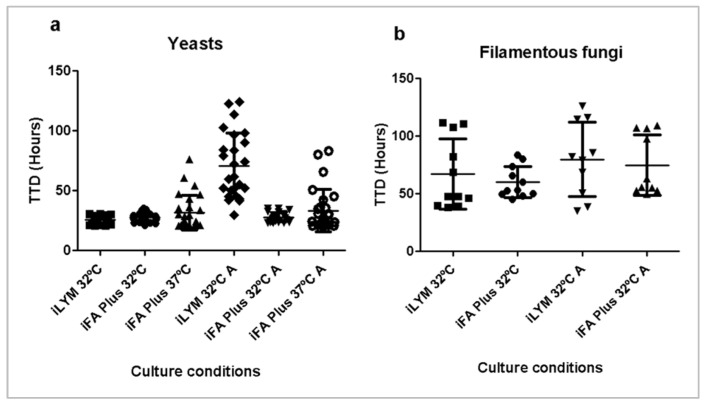
Time to detection in hours for 3 replicates of (**a**) 10 yeasts and (**b**) 8 filamentous fungi in different culture bottles and incubation temperatures, with or without amphotericin B (A). BACT/ALERT^®^ bottles containing Corneal Chamber media were inoculated with <100 CFU of each microorganism. Filamentous fungi at 37 °C are not plotted because 6 out of 8 failed to grow.

Aggregated TTD across replicates are presented in Figure 4, and detection rates are reported in Table 3.

#### 3.2.1. Effect of Incubation Temperature

Fungal isolates in iFA Plus bottles were significantly more likely to be detected by BACT/ALERT^®^ at the lower incubation temperature of 32 °C (94.4%) compared with 37 °C (61.1%, *p* < 0.0001), regardless of amphotericin B supplementation. This effect was primarily driven by filamentous fungi, with only 25% detected at 37 °C versus 87.5% at 32 °C (*p* < 0.0001). When considering only yeasts, the effect of temperature was not significant (*p* > 0.05). Regarding TTD, among all isolates that were detected, there was no significant difference between temperatures (*p* > 0.05): 31.4 ± 9.4 h at 32 °C versus 33.6 ± 13.9 h at 37 °C without amphotericin B, and 34.0 ± 16.6 h at 32 °C versus 35.4 ± 17.0 h at 37 °C with amphotericin B.

#### 3.2.2. Effect of Culture Bottle Type

When comparing culture media at 32 °C without amphotericin B, iFA Plus bottles detected a higher percentage of fungal isolates than iLYM bottles (94.4% vs. 77.8%, *p* = 0.0235). This difference was even more pronounced when amphotericin B was present in the corneal media (94.4% vs. 72.2%, *p* = 0.0036). TTD for all the isolates that were detected did not differ significantly between iLYM and iFA Plus bottles at 32 °C without amphotericin B (iLYM: 37.5 ± 24.9 h vs. iFA Plus: 36.8 ± 16.9 h). However, with amphotericin B supplementation, TTD was significantly shorter in iFA Plus bottles (iLYM: 73.2 ± 28.7 h vs. iFA Plus: 40.4 ± 25.2 h; *p* < 0.0001). This notable difference is attributed to the presence of neutralizing beads in iFA Plus bottles.

#### 3.2.3. Effect of Amphotericin B

Due to the presence of these beads, supplementation of Corneal Chamber media with amphotericin B had no significant effect on fungal detection in iFA Plus bottles at either 32 °C or 37 °C. In contrast, amphotericin B caused a reduction in fungal detection when iLYM bottles were used (77.8% without amphotericin B vs. 72.2% with amphotericin B), although this difference did not reach statistical significance. However, the effect of amphotericin B was clearly observed in TTD for the isolates that were detected. TTD was significantly longer in iLYM bottles at 32 °C with amphotericin B (73.2 ± 28.7 h) than in iLYM bottles at 32 °C without amphotericin B (37.5 ± 25.0 h; *p* < 0.0001).

#### 3.2.4. Overall Results

**Figure 5:**
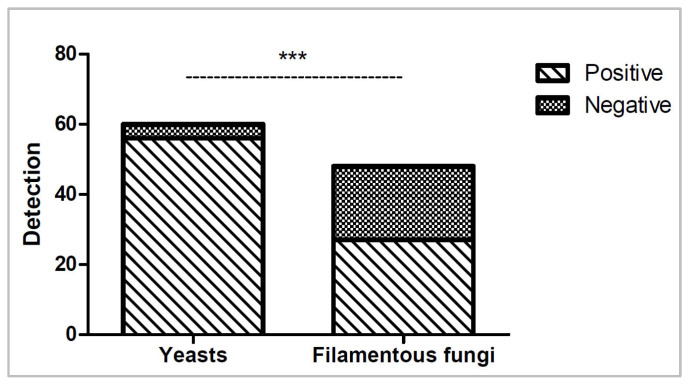
Number of yeasts and filamentous fungi isolates detected (positive) or not detected (negative) across all six culture conditions. Statistical significance (*p* < 0.0001) is shown as ***.

All fungal isolates were able to grow on TSA and SDA plates within five days, with yeasts incubated at 32 °C and filamentous fungi incubated at 22 °C; however, considerable variability was observed across isolates under different automated culture conditions. Overall, BACT/ALERT^®^ performed better for yeast detection (56/60, 93.3%) across all conditions than for filamentous fungi detection (27/48, 56.2%; *p* < 0.0001), as shown in Figure 5. Nine out of ten (90%) yeast isolates were detected under all conditions, whereas only two out of eight (25%) filamentous fungi isolates were detected under all conditions. Only one, *Cladosporium sphaerospermum*, failed detection in all culture conditions and was unable to grow at 32 °C, although it did grow at 22 °C. 

No isolate was detected solely in iLYM bottles at 32 °C, whereas *Debaryomyces hansenii*, *Penicillium chrysogenum*, and *Stachybotrys chlorohalonata* were detected only in iFA Plus bottles at 32 °C, with or without amphotericin B. Consequently, a two-bottle approach combining iFA Plus at 37 °C and iLYM at 32 °C did not improve detection.

**Figure 6:**
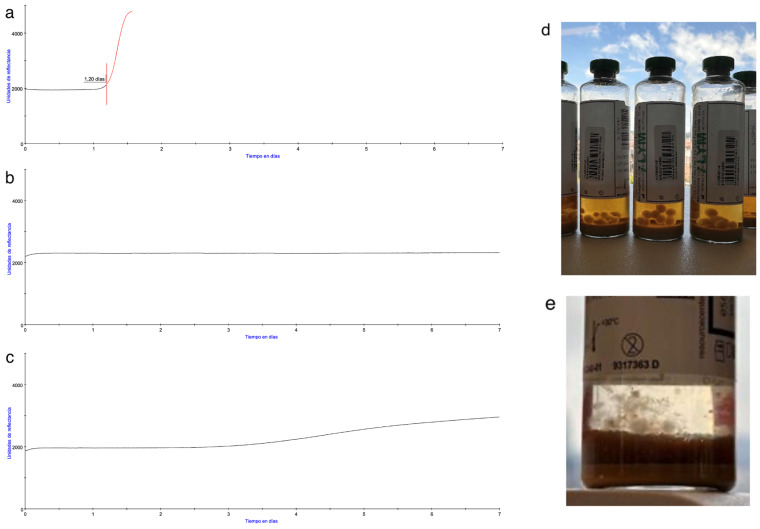
On the left, comparison of BACT/ALERT^®^ growth curves from (**a**) Positive bottle, (**b**) Negative bottle, and (**c**) Negative bottle which was positive by post-incubation visual inspection. On the right, iLYM vented bottles, after removal from the system, with visible growth of *Curvularia* sp. (**d**) and *Penicillium chrysogenum* (**e**) which was not detected by the system.

Importantly, some fungal isolates in iLYM bottles were detected only by visual inspection at the end of incubation, as the system itself failed to detect them as shown in Figure 6.

Overall, iFA Plus bottles incubated at 32 °C demonstrated superior detection performance for all fungal isolates, regardless of amphotericin B supplementation.

## 4. Discussion

Fungal infections post-keratoplasty are rare but serious complications that can significantly impact the success of the graft and the patient’s vision. Their clinical importance lies in their insidious onset, diagnostic difficulty, resistance to treatment, and potential to cause graft failure or intraocular infection. Among other factors, contaminated donor corneal tissue may be the origin of the infection and therefore it is a challenge for eye banks to promptly detect contaminated corneas and discard them. Fungal detection in microbiology is challenging due to the slow and variable growth of fungi and the need for specialized media and techniques. Yeasts, which are unicellular organisms, can be assimilated to bacteria, but filamentous fungi, which are multicellular, have differential characteristics. To start with, since they grow attached to the cornea surface or forming fungal balls, it is difficult to extract a representative sample from the corneal medium to be cultured. Furthermore, special media and incubation temperatures are needed to promote their growth.

The results of this study confirmed the high sensitivity and specificity of the BACT/ALERT^®^ system reported in the literature for sterility testing, supporting its use as a robust and reliable platform [37,38,46]. We also validated the suitability of the method with iFA Plus bottles for microbiological monitoring of 6 corneal preservation media, even though these matrices contain antibiotics and antifungals. By contrast, the BACTEC system with BD BACTEC Plus Aerobic/F and Anaerobic/F bottles did not suport the growth of *Bacillus subtilis* and produced a delayed detection of all microorganisms in the presence of corneal media [47]. This problem could only be surmounted when a special device was used to neutralize the antimicrobials before incubation in BACTEC [48], which implied a higher manual workload.

Other reports have highlighted that certain slow-growing or fastidious fungi may escape detection under standard clinical algorithms [39,49], emphasizing the need to optimize culture conditions. Building on this, our work focuses on improving fungal detection by evaluating how culture performance is influenced by incubation temperature, bottle type, and the addition of amphotericin B as an antifungal agent in the preservation medium.

Incubation temperature was shown to exert a decisive influence on fungal detection. The results clearly indicate that 32 °C provides more favorable conditions for the growth of a broad range of environmental yeasts and filamentous fungi commonly encountered in corneal preservation media, as reported in multiple studies [29,39]. Physiologically, many filamentous fungi exhibit reduced enzymatic activity and impaired membrane stability at temperatures approaching 37 °C [50], which explains the failure of tested species such as *Alternaria alternata*, *Arthrinium arundinis*, *Stachybotrys chlorohalonata*, *Cladosporium sphaerospermum*, *Penicillium chrysogenum*, and *Curvularia* sp. to grow under higher-temperature conditions. On the other hand, yeasts show broader temperature tolerance, although our results confirm that species such as *Candida albicans* ATCC 10231 and *Wickerhamomyces anomalus* proliferate more slowly at 37 °C, whereas *Debaryomyces hansenii* failed to grow at 37 °C, in agreement with previous reports highlighting the temperature-dependent growth limitations of this species [51].

Although our study compared incubation at 32 °C and 37 °C, other reports have suggested that lower, ambient-like temperatures of 20 °C to 25 °C may further enhance the recovery of both filamentous fungi and certain yeasts [52]. Indeed, environmental monitoring studies have demonstrated that incubation at 22 °C increased filamentous fungi yields, with some authors reporting maximal recovery at 22.5 °C [53]. However, such conditions markedly delay the growth of the bacterial test strains required by current Ph. Eur. Standards [54], which makes this approach unsuitable for sterility testing of corneal preservation media. By contrast, incubation at 32 °C offers a practical compromise, as it enhances fungal recovery while still allowing early detection of bacteria. Nevertheless, our findings corroborate previous studies indicating that anaerobes, particularly *Cutibacterium acnes*, grow better at higher temperatures [55], since this species was detected almost one day later at 32 °C than at 37 °C. This observation highlights the trade-off between optimal fungal recovery and timely bacterial detection, suggesting that adopting dual incubation strategies at both temperatures could offer a more balanced and comprehensive approach.

The choice of culture bottle was another key determinant of performance. Contrary to the initial expectation of greater sensitivity based on published results [29], vented iLYM bottles performed worse than iFA Plus bottles. Although fungal growth of most species was slightly faster in iLYM bottles, the overall detection rate was significantly higher in iFA Plus bottles. The poorer performance of iLYM was particularly evident for *Debaryomyces hansenii*, *Stachybotrys chlorohalonata*, and *Cladosporium sphaerospermum*, which were not detected. Specifically, the latter species was not recovered under any automated condition and only grew on SDA plates at 22 °C, consistently with its known requirement for lower incubation temperatures [56,57]. However, it has to be noted that *Cladosporium sphaerospermum*, although recognized as an etiological agent of post-traumatic keratitis, it is rarely clinically relevant in corneal transplantation [58], and in our microbiological series it was only detected once, adhering to the bottle surface rather than on the cornea or actively proliferating in the medium.

Since there is a growing interest in the addition of antifungals in corneal preservation media [30], we studied the effect of amphotericin B, at a concentration of 2.5 μg/mL, in fungal detection. This concentration was chosen as it represents the highest level commonly used in corneal storage media, being effective against fungi while remaining non-toxic to endothelial cells [9,59]. Our results prove that the presence of amphotericin B in corneal preservation media has a substantial effect on detection, depending on the bottle type. In iLYM bottles, which lack neutralizing beads, TTD was significantly delayed in antifungal-containing media compared to media without amphotericin B for all species, except *Aspergillus brasiliensis* ATCC 16404. Furthermore, in the case of *Pichia kudriavzevii*, growth was observed in only one of three replicates in iLYM bottles containing amphotericin B. These findings indicate that amphotericin B hinders fungal growth and detection in these bottles, in accordance with previous reports on its effectiveness against fungi [32,60]. In contrast, iFA Plus bottles fully mitigated the antifungal effect due to their neutralizing beads: TTD was comparable in corneal preservation media with or without amphotericin B, with no statistically significant differences for any tested species. This confirms previous reports indicating that the beads effectively inactivate amphotericin B [61,62], preventing false negatives or delayed signals [63], which is particularly critical in eye banks. It is a highly relevant result, as the use of antifungals in preservation media is intended to reduce contamination risk, yet it can mask contamination in culture systems without neutralization capability.

Beyond detection rates, several practical limitations of iLYM bottles became evident. Their requirement for venting prior to use, as recommended in the user guide [45], increases the risk of contamination, while the absence of neutralizing beads delay fungal detection in the presence of antifungals. Furthermore, some species, including *Curvularia* sp. and *Penicillium chrysogenum*, were completely undetected by the BACT/ALERT^®^ system despite visible hyphal growth once the bottles were removed. This highlights a featured limitation noted in previous studies: the clinical detection algorithm, which relies on more pronounced growth slopes, may fail to detect slow-growing filamentous fungi with less steep growth curves [39,64]. Although a change in algorithm could potentially improve sensitivity, it was not adopted here to avoid false positives and the unnecessary rejection of transplantable tissues. These findings underscore the importance of post-incubation visual inspection of culture bottles as an additional safeguard in tissue banks, as some reports recommend [65,66].

In summary, the present study demonstrates that BACT/ALERT^®^ is a reliable automated plattform for the sterility testing of corneal preservation media, even if they contain antimicrobials. Incubation temperature and culture bottles are critical and should be tailored to the intended purpose. iFA Plus bottles incubated at 32 °C showed superior detection performance for all fungal isolates, both collection and clinical strains, regardless of amphotericin B supplementation of corneal preservation media. iLYM bottles, not only detected a lower percentage of fungal isolates than iFA Plus bottles at 32 °C, but also had practical limitations that preclude their suitability for sterility testing of corneal preservation media with antimicrobials. iFA Plus bottles incubated at 32 °C detected 94.4% (17 out of 18) fungal isolates, including the most frequently found in corneas such as *Candida* spp. and *Aspergillus* spp.

Nevertheless, some limitations of the present study should be noted. Although multiple real life isolates were included (18 out of 21 diferent species recovered in routine cultures during 16 months), expanding the panel of tested strains would improve the robustness of the findings, and increasing the number of replicates could further help confirm reproducibility. Future studies might explore algorithmic improvements in automated systems or complementary approaches, such as PCR-based assays, to further enhance fungal detection.

We proved that adjusting incubation temperatures in growth-based automated systems—a strategy fully permitted by the Ph. Eur. for sterility testing—enhances environmental fungal detection in corneal preservation media. Therefore, shifting iFA Plus bottles to 32 °C for improved fungal recovery, while maintaining iFN Plus at 37 °C for timely anaerobic bacterial detection, is a worthwhile strategy for sterility testing of donor corneas. Furthermore, it does not increase the cost, since no more bottles are added. Nonetheless, fungi that only grow below 30 °C may still escape detection, although their clinical relevance remains to be determined, highlighting the value of complementary methodologies such as visual inspection of the corneal media to further improve sensitivity.

## 5. Conclusions

The present study shows that incubation conditions critically influence fungal detection in corneal preservation media when using the BACT/ALERT^®^ system. iFA Plus bottles incubated at 32 °C provided the highest detection rates, effectively neutralizing the inhibitory effect of amphotericin B and enabling reliable recovery of both yeasts and filamentous fungi, except for *Cladosporium sphaerospermum*. In contrast, incubation at 37 °C and the use of iLYM bottles markedly reduced sensitivity and led to delayed or missed detections, particularly for filamentous fungi.

Overall, the findings support incubating iFA Plus bottles at 32 °C for optimal fungal recovery, while maintaining iFN Plus at 37 °C to allow timely detection of anaerobic bacteria. This balanced strategy, in agreement with Ph. Eur. requirements, enhances fungal detection without compromising bacterial growth and is optimal for sterility testing of corneal storage media.

## Figures and Tables

**Table 1:** Reproducibility. Mean, standard deviation (SD), and coefficient of variation (CV) of TTD (in hours) from six independent experiments. The inoculated colony-forming units (CFU) are presented as mean ± SD.

Microorganism	CFU Inoculated (Mean ± SD)	Mean (Hours)	SD	CV (%)
*Staphylococcus aureus*	31.7 ± 13.8	15.8	0.4	2.6
*Pseudomonas aeruginosa*	41.0 ± 12.1	16.7	1.3	7.6
*Bacillus subtilis* subsp. *spizizenii*	45.2 ± 9.9	12.4	0.4	3.6
*Candida albicans*	51.2 ± 18.4	46.4	2.5	5.4
*Aspergillus brasiliensis*	38.0 ± 6.6	49.5	4.5	9.0
*Streptococcus pyogenes*	25.3 ± 2.5	14.1	0.4	2.9
*Micrococcus luteus*	19.7 ± 2.5	34.8	3.3	9.5
*Clostridium sporogenes*	22.3 ± 15.4	17.3	0.7	4.1
*Cutibacterium acnes*	49.5 ± 14.5	129.3	16.9	13.1
*Enterobacter aerogenes*	24.4 ± 2.4	12.4	0.1	1.1
*Staphylococcus epidermidis*	24.8 ± 1.6	18.2	0.4	2.5

**Table 2:** Time to detection in hours (mean of three replicates) for (**a**) yeasts and (**b**) filamentous fungi in different culture bottles and incubation temperatures, with or without amphotericin B (A). Color coding: green: ≤ 2 days; yellow: 3–4 days; red > 4 days; purple: negative. Abbreviations: NEG = negative; VIS POS = positive detected by post-incubation visual inspection, not detected by BACT/ALERT^®^ (bioMérieux, Marcy-l’Étoile, France); CFU = Colony Forming Units.

Microorganism		Corneal Chamber			Corneal Chamber + A		
(**a**) Yeasts	**CFU**	**iLYM 32 °C**	**iFA Plus 32 °C**	**iFA Plus 37 °C**	**iLYM 32 °C**	**iFA Plus 32 °C**	**iFA Plus 37 °C**
*Candida albicans* ATCC	23	25.4	29.9	45.6	114.3	29.4	45.8
*Candida albicans*	29	24.7	31.0	29.2	76.4	31.8	30.2
*Candida tropicalis*	15	20.7	22.3	20.6	42.2	23.2	21.0
*Candida parapsilosis*	17	30.1	34.2	34.9	54.5	34.6	35.4
*Candida glabrata*	32	23.6	24.7	20.9	90.7	24.6	20.8
*Clavispora lusitaniae*	37	28.9	25.4	22.9	61.9	25.7	23.0
*Pichia norvegensis*	13	24.9	27.0	24.0	43.6	26.9	24.3
*Pichia kudriavzevii*	39	21.5	22.9	21.4	44.9	23.4	21.7
*Wickerhamomyces anomalus*	52	29.9	28.9	63.6	90.2	29.4	76.1
*Debaryomyces hansenii*	15	NEG	121.2	NEG	NEG	112.2	NEG
(**b**) Filamentous fungi							
*Aspergillus brasiliensis* ATCC	17	38.8	49.1	44.0	41.3	53.3	45.8
*Aspergillus fumigatus*	24	45.8	48.0	40.1	82.2	71.3	45.1
*Penicillium chrysogenum*	33	VIS POS	111.1	NEG	VIS POS	94.9	NEG
*Arthrinium arundinis*	10	78.9	109.8	NEG	118.9	103.6	NEG
*Cladosporium sphaerospermum*	25	NEG	NEG	NEG	NEG	NEG	NEG
*Stachybotrys chlorohalonata*	24	NEG	88.1	NEG	NEG	72.6	NEG
*Alternaria alternata*	12	82.2	60	NEG	68.6	62.9	NEG
*Curvularia* sp.	16	68.4	65.3	NEG	VIS POS	63.1	NEG

**Table 3:** Percentage (%) of fungal isolates detected under each culture condition. Abbreviations: A = Amphotericin B.

Fungi	Corneal Chamber			Corneal Chamber + A		
	iLYM 32 °C	iFA Plus 32 °C	iFA Plus 37 °C	iLYM 32 °C	iFA Plus 32 °C	iFA Plus 37 °C
Yeasts	90.0	100.0	90.0	90.0	100.0	90.0
Filamentous fungi	62.5	87.5	25.0	50.0	87.5	25.0
Total	77.8	94.4	61.1	72.2	94.4	61.1

## Data Availability

The original contributions presented in this study are included in the article/Supplementary Material. Further inquiries can be directed to the corresponding authors.

## Supplementary Materials

The following supporting information can be downloaded at: https://www.mdpi.com/article/10.3390/microorganisms13112562/s1, Figure S1: Mean time to detection (TTD) of 18 microorganisms tested (10 yeasts and 8 filamentous fungi) using the BacT/ALERT^®^ system under different conditions. Statistical significance is denoted in figures as: * (*p* ≤ 0.05), ** (*p* ≤ 0.01), *** (*p* ≤ 0.001). Non‐significant differences are shown as ns (p > 0.05), while NA denotes cases where statistical analysis was not applicable due to absence of replicates. In addition, no statistical data are shown for *Alternaria alternata* and *Curvularia* sp. because no replicates were tested. Abbreviations: A = Amphotericin B; (!) = For *Pichia kudriavzevii* indicates that only one of the three replicates showed growth under these conditions, and for *Penicillium chrysogenum* and *Curvularia* sp., although the system reported a negative result, hyphal growth was observed during post‐incubation visual inspection.

## Abbreviations

The following abbreviations are used in this manuscript:

Ph. Eur.	European Pharmacopoeia
CFU	Colony-Forming Units
A	Amphotericin B
TTD	Time to Detection
