# Supplementary material for: Comparative Evaluation of Temperature, Bottle Type, and Amphotericin B on Fungal Detection in Corneal Preservation Media Using the BACT/ALERT System®
Source: Microorganisms. 2025 Nov 10;13(11):2562. doi: 10.3390/microorganisms13112562 (PMC12654506; doi:10.3390/microorganisms13112562)
Supplement: Supplementary file 1 [file microorganisms-13-02562-s001.zip › microorganisms-3937596-supplementary.pdf]

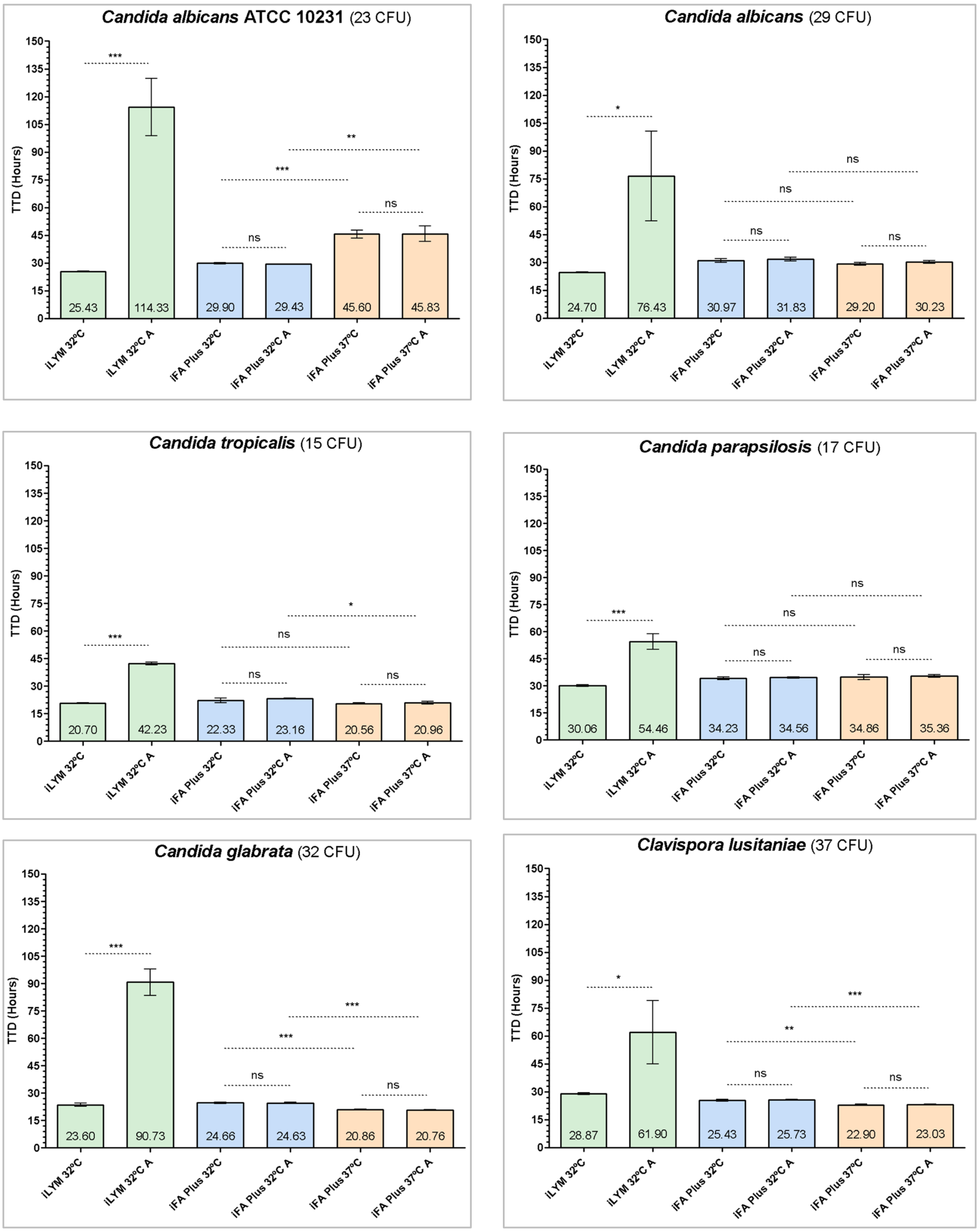
Candida albicans ATCC 10231 (23 CFU)
TTD (Hours)
25.43
114.33
29.90
29.43
45.60
45.83
iLYM 32ºC
iLYM 32ºC A
iFA Plus 32ºC
iFA Plus 32ºC A
iFA Plus 37ºC
iFA Plus 37ºC A
Candida albicans (29 CFU)
24.70
76.43
30.97
31.83
29.20
30.23
Candida tropicalis (15 CFU)
20.70
42.23
22.33
23.16
20.56
20.96
Candida parapsilosis (17 CFU)
30.06
54.46
34.23
34.56
34.86
35.36
Candida glabrata (32 CFU)
23.60
90.73
24.66
24.63
20.86
20.76
Clavispora lusitaniae (37 CFU)
28.87
61.90
25.43
25.73
22.90
23.03
ns

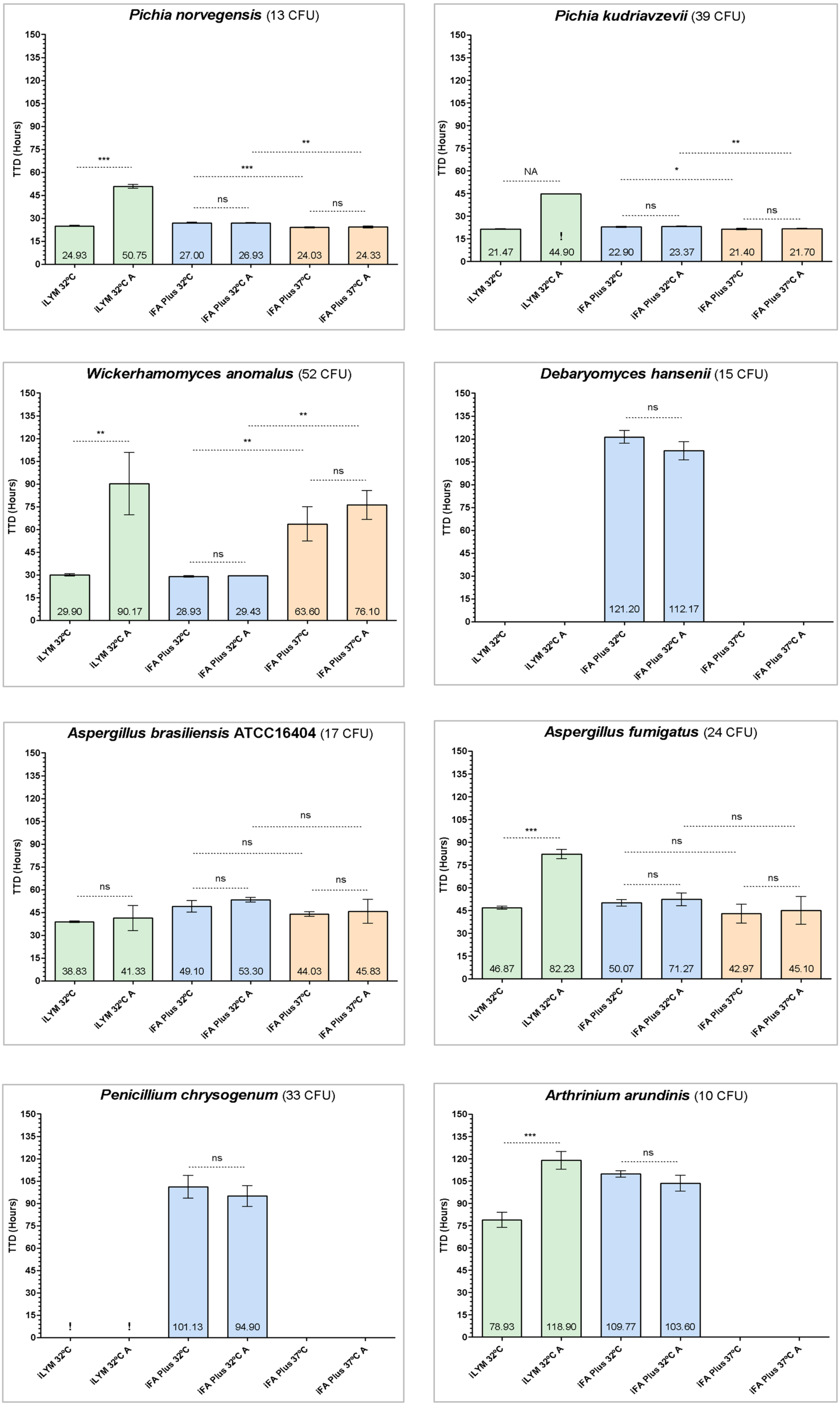
Pichia norvegensis (13 CFU)
TTD (Hours)
iLYM 32ºC
iLYM 32ºC A
iFA Plus 32ºC
iFA Plus 32ºC A
iFA Plus 37ºC
iFA Plus 37ºC A
24.93
50.75
27.00
26.93
24.03
24.33
***
***
**
ns
ns
Pichia kudriavzevii (39 CFU)
NA
*
**
ns
ns
!
21.47
44.90
22.90
23.37
21.40
21.70
Wickerhamomyces anomalus (52 CFU)
**
**
**
ns
ns
29.90
90.17
28.93
29.43
63.60
76.10
Debaryomyces hansenii (15 CFU)
ns
121.20
112.17
Aspergillus brasiliensis ATCC16404 (17 CFU)
ns
ns
ns
ns
ns
38.83
41.33
49.10
53.30
44.03
45.83
Aspergillus fumigatus (24 CFU)
***
ns
ns
ns
ns
46.87
82.23
50.07
71.27
42.97
45.10
Penicillium chrysogenum (33 CFU)
ns
!
!
101.13
94.90
Arthrinium arundinis (10 CFU)
***
ns
78.93
118.90
109.77
103.60

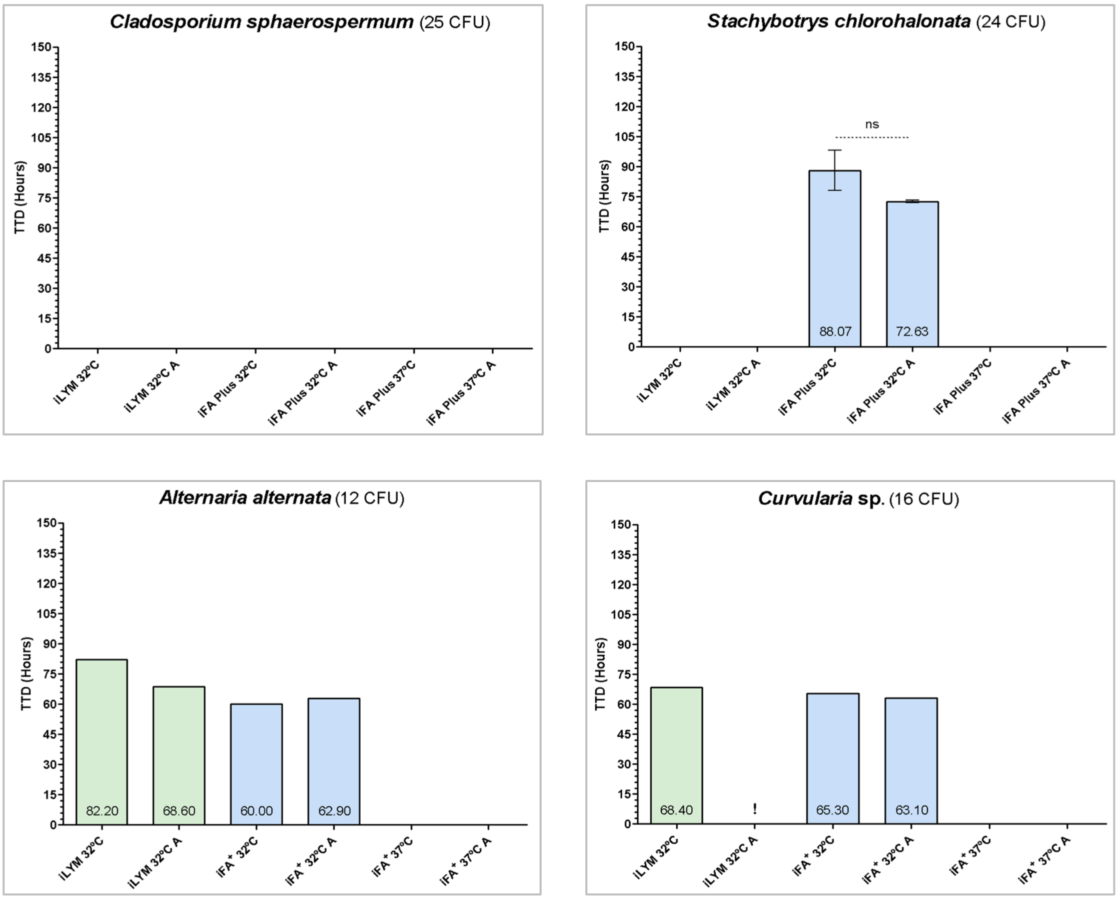

Supplementary Figure S1. Mean time to detection (TTD) of 18 microorganisms tested (10 yeasts and 8 filamentous fungi) using the BacT/ALERT® system under different conditions. Statistical significance is denoted in figures as: * ($p \leq 0.05$), ** ($p \leq 0.01$), *** ($p \leq 0.001$). Non-significant differences are shown as ns ($p > 0.05$), while NA denotes cases where statistical analysis was not applicable due to absence of replicates. In addition, no statistical data are shown for *Alternaria alternata* and *Curvularia* sp. because no replicates were tested. Abbreviations: A = Amphotericin B; (!) = For *Pichia kudriavzevii* indicates that only one of the three replicates showed growth under these conditions, and for *Penicillium chrysogenum* and *Curvularia* sp., although the system reported a negative result, hyphal growth was observed during post-incubation visual inspection.
